# Predictors of 30-day hospitalization in patients with worsening heart failure receiving outpatient intravenous diuretics

**DOI:** 10.1371/journal.pone.0342263

**Published:** 2026-02-17

**Authors:** Willemijn A. van Maarschalkerwaart, Eric Wierda, Dominique de Boer, Nini H. Jonkman, Eric Boersma, Jasper J. Brugts, Loek van Heerebeek

**Affiliations:** 1 Department of Cardiology, OLVG, Amsterdam, The Netherlands; 2 Department of Cardiology, Dijklander Ziekenhuis, Hoorn, The Netherlands; 3 Department of Research and Epidemiology, OLVG, Amsterdam, The Netherlands; 4 Department of Cardiology, Thorax Center, Cardiovascular Institute, Erasmus MC, Rotterdam, The Netherlands; University of Naples Federico II, ITALY

## Abstract

**Background:**

Outpatient intravenous (IV) diuretic treatment is an effective and safe strategy for worsening heart failure (WHF). Still, hospitalization cannot be avoided in a substantial portion of patients and potential predictors of HF hospitalization (HFH) are eagerly awaited.

**Aim:**

We aimed to identify predictors of HFH after outpatient IV diuretic treatment for WHF, in order to improve the selection of patients who qualify for successful outpatient IV diuretic treatment.

**Methods and results:**

We studied WHF patients receiving intravenous diuretics in an outpatient day-care setting in one of two Dutch hospitals. A total of 366 patients from hospital A were used to identify predictors of 30-day HF (re-)hospitalization (HFH), which occurred in 88 (24.0%). Mean age was 76 years, 57% were male and 49% had ejection fraction below 40%. Age, eGFR, NT-proBNP, sodium, and haemoglobin were identified as predictors of HFH. The multivariable logistic regression model containing these factors had acceptable calibration and discrimination (AUC 0.73). The performance of the model was less favorable in the 127 patients from hospital B (29 patients with 30-day HFH), with AUC 0.65 and suboptimal calibration, indicating overestimation of risk. Doubling of NT-proBNP plasma levels and higher ambulatory oral loop diuretic dosages were strong predictors of mortality and HFH at 6 months in hospital A.

**Conclusion:**

In patients with WHF receiving outpatient day-care intravenous diuretic treatment, age, eGFR, NT-proBNP, sodium and haemoglobin predicted 30-day HFH. These factors may guide decisions on day-care treatment versus hospitalization, but require further validation Fig 2.

## Introduction

Outpatient treatment strategies for episodes of worsening heart failure (WHF) – sometimes referred to as ambulatory treatment – have gained increased interest as a safe alternative to in-hospital treatment to reduce the burden on the hospital wards [1]. Different multidisciplinary units involving heart failure (HF) nurses and cardiologists have been studied, predominantly in USA settings, but also in the European setting [2–6]. Outpatient treatment typically consists of one or multiple sessions of intravenous (IV) diuretic treatment during a period of three to six hours in patients with signs and symptoms of congestion and who are unresponsive to intensification of oral diuretics [7].

While this outpatient strategy is safe, reported rehospitalization rates after such treatment range from 9–42% [3,8]. This broad range likely reflects differences in patient selection, treatment protocols, and hospital’s logistic processes. To guide selection, Girerd et al. formulated factors in favor of and against treatment with IV diuretics in an outpatient setting. Factors in favor include acceptable vital parameters (i.e., systolic blood pressure > 100 mmHg, heart rate between 50–120 bpm, oxygen saturation > 92%), whereas presence of a critical trigger (i.e., arrhythmia, acute coronary syndrome), NYHA class IV, severely impaired renal function or electrolyte disturbances are considered factors against outpatient treatment. Using these factors for patient selection can aid in reducing healthcare expenses and lower the strain on the hospitals’ admission capacities [7].

However, despite implementing these recommendations in the inclusion and exclusion criteria for our outpatient clinic, the 30-day HF hospitalization rate in these HF patients remains as high as 31% [3]. These high hospitalization rates after outpatient IV diuretic treatment underscore the unmet need for a better selection strategy to identify which WHF patients would benefit more from direct in-hospital treatment instead of the outpatient strategy.

Current available evidence on prognostic factors specific to the WHF population treated in an outpatient setting is scarce and limited by sample size (n = 259 and n = 107) and study design [3,9]. Furthermore, existing risk models to predict HF hospitalization risk, such as the Barcelona Bio-HF, are developed and validated in cohorts including stable ambulatory HF patients and are not tailored to the WHF population [10,11].

Selecting the appropriate patients for outpatient treatment with IV diuretics informs caregivers and patients on the decision for direct in-hospital or outpatient treatment. Against this background, we aimed to identify prognostic factors that are associated with a high risk of HF (re-)admission within 30 days after the index presentation. Secondary endpoints include repeated outpatient treatment visits, HF (re-)hospitalization and mortality within six months.

## Methods

### Patient cohorts

This was an investigator-initiated observational retrospective cohort study including all patients treated with IV diuretics at the outpatient clinic of two hospitals in the Netherlands. The primary cohort consisted of patients from a large urban teaching hospital (hospital A) treated between November 2018 and December 2022 (n = 366) and was used to identify predictors of HF hospitalization. The second cohort consisted of patients from a regional hospital located in a semi-urban area (hospital B), treated between November 2018 and June 2023 and was used to validate these predictors.

Inclusion criteria for treatment at the outpatient clinics in both hospitals were in line with recommendations from Girerd et al 2022 [7]: an established diagnosis of chronic HF for at least three months with symptoms of WHF, unresponsive to intensification of oral medication or too symptomatic to treat at home. Furthermore, patients with newly onset HF, ischemic heart disease with current ischemia, valve disease who are awaiting intervention, new cardiac rhythm disturbances, or cardiogenic shock or respiratory failure at time of presentation were excluded from outpatient treatment. Patients were excluded from this study if six-month follow-up data on the study endpoints were not available. A flowchart of patient inclusion in the different cohorts is shown in S1 Fig in S1 File. The outpatient IV diuretic treatment regimen was the same for both hospitals and is shown in Table 1 [3].

**Table 1:** Outpatient treatment regimen used in Hospital A and Hospital B.

Home dosage of loop diuretics	IV furosemide dosage	
	Bolus	Perfusor
40 mg furosemide or equivalent^*^	80 mg	none
80 mg furosemide or equivalent^*^	80 mg	120 mg in 4 hours
120 mg furosemide or equivalent^*^	none	240 mg in 4 hours
> 240 mg furosemide or equivalent^*^	80 mg	240 mg in 4 hours

IV, intravenous. * 40 mg furosemide is equivalent to 1 mg bumetanide. Adapted from Wierda et al., 2023.

This study was conducted in accordance with the Declaration of Helsinki [12]. The research protocol was approved by the institutional review board of the participating hospitals (WO.23.164). Written informed consent was obtained from participants for the use of their medical data in this study where possible. In cases where patients were deceased at time of data collection or where a language barrier prevented informed consent, data were included in accordance with the approval of the institutional review board, which waived the requirement for informed consent for these specific cases.

### Data collection

Data on baseline patient characteristics, laboratory values, and medical treatment were collected based on electronic patient records and entered into standardized online data collection forms. Based on previous studies [10,11], availability in the cohorts and ease of use in routine clinical practice, the following baseline variables were considered as potential predictors: age, sex, BMI, previous HF hospitalization, dosage oral loop diuretics, type of HF (heart failure with reduced ejection fraction [HFrEF], heart failure with mildly reduced ejection fraction [HFmrEF], heart failure with preserved ejection fraction [HFpEF]), kidney function, NT-proBNP, sodium, haemoglobin, presence of Cardiac Resynchronization Therapy Defibrillator or Pacemaker (CRT-D or CRT-P), treatment with angiotensin-converting enzyme (ACE)-inhibitor, angiotensin receptor blocker (ARB), or angiotensin receptor blocker-neprilysin inhibitor (ARNI), and language barrier.

Dosage of oral loop diuretics was defined as the home dosage of furosemide or furosemide equivalent (with 1 mg bumetanide being equivalent to 40 mg furosemide) and categorized as low (0–80 mg furosemide or equivalent), middle (81–160 mg furosemide or equivalent), or high (>160 mg furosemide or equivalent). Kidney function was based on the eGFR (CKD-EPI formula) value at time of first outpatient treatment, with chronic kidney disease defined as an eGFR below 60 mL/min/1.73m^2^ for at least 3 months. A detailed description of all predictor variables and the used definitions is provided in S1 Table in S1 File of the supporting information.

Data were accessed for research purposes between 27 February and 10 July 2024. During data collection, designated authors had access to a secured subject identification code list in order to extract data from the electronic health records. Data were pseudonymized at time of entry into the secured Castor EDC system, and only pseudonymized data were used for statistical analysis.

### Endpoints

The primary endpoint was defined as HF (re-)hospitalization within 30 days after the index event. Heart failure (re-)hospitalization was defined as more than 24 hours in-hospital stay caused by WHF symptoms and/or signs requiring administration of IV diuretics. Secondary endpoints included six-month HF (re-)hospitalization, repeated outpatient treatment, and mortality. Repeated outpatient treatment was defined as two or more outpatient treatments with IV diuretics after the first outpatient treatment.

### Statistical analysis

Baseline characteristics were summarized using frequencies and percentages for categorical variables and mean with standard deviation (SD) or median with interquartile range (IQR) for continuous and count variables. Statistical differences in patients with and without a 30-day HF (re-)hospitalization and comparisons between the cohorts from hospital A and hospital B were conducted using the χ2 test, unpaired t-test, or Mann-Whitney U test as appropriate.

The Transparent Reporting of a multivariable prediction model for Individual Prognosis Or Diagnosis (TRIPOD) was used as a guideline for developing and validating our prediction model for the primary endpoint [13].

Multivariable logistic regression with a stepwise backward procedure was used to identify predictors of the primary endpoint in patients from hospital A. The potential predictors were checked on multicollinearity using variance inflation factors (VIF), with VIF > 5 considered indicative of problematic multicollinearity. Additionally we used Spearman correlations, with r ≥ 0.8 as an indication for multicollinearity. No multicollinearity was identified (S2 and S3 Tables in S1 File). Missing values were limited to one predictor variable (0.5% missing) in hospital A and two predictors in hospital B (1.5% missing) and were imputed using multiple imputation by chained equations in a single imputed dataset.

The Akaike’s information criterion (AIC) was used for model selection and reduction of redundant variables. Bootstrapping techniques (250 bootstrap samples) were used to internally validate the set of predictors and address the possibility of overfitting by calculating shrinkage factors. We multiplied the shrinkage factors with the original coefficients from the identified set of predictors and fitted a new intercept to maintain overall calibration of the model. The final set of predictors was presented with the odds ratios (OR) and 95% CI’s.

An exploratory external validation of the identified predictors was performed in patients from hospital B. We fitted the shrunken coefficients from the set of predictors in the cohort from hospital B. Calibration, Area Under the Receiver Operating Curve (AUROC) and R^2^ were used to assess performance of the set of predictors from hospital A in hospital B.

Independent predictors for the secondary endpoints were analyzed in a similar fashion using a logistic regression model with stepwise backward procedure. As these were exploratory analysis, we did not perform internal and external validation for the secondary endpoints.

For predictors of the primary endpoint, a sensitivity analysis was performed on patients with HFrEF, HFmrEF, and HFpEF separately in hospital A’s dataset, to assess robustness of identified predictors across subgroups relevant for clinical practice.

Data were analyzed using R version 4.4.0 with the ‘rms’, ‘pROC’ and ‘mice’ packages. P-values of <0.05 were considered statistically significant.

## Results

A total of 366 consecutive patients from hospital A were included, of whom 88 (24.0%) experienced a 30-day HF (re-)hospitalization. Their baseline characteristics are presented in Table 2, whereas diuretic use during outpatient treatment is described in Table 3.

**Table 2:** Baseline characteristics of hospital A, stratified by endpoint of HF (re-)hospitalization within 30 days (n = 366).

	Overall n = 366	No HFH within 30 days n = 278	HFH within 30 days n = 88	P-value
**Age, years**	76.1 (10.1)	76.8 (10.0)	73.8 (9.9)	0.012
**Male**	207 (56.6)	156 (56.1)	51 (58.0)	0.86
**BMI, kg/m** ^**2**^	29.7 (6.8)	29.6 (6.8)	29.9 (6.6)	0.70
**Language barrier**	81 (22.1)	57 (20.5)	24 (27.3)	0.24
**HF hospitalization within previous 12 months**	270 (73.8)	198 (71.2)	72 (81.8)	0.067
**Number of HF hospitalizations in previous year**	2.0 [1.0, 2.0]	2.0 [1.0, 2.0]	2.0 [2.0, 2.0]	0.049
**HF duration in months**	47.5 [16.0, 116.5]	47.0 [17.2, 110.0]	50.0 [9.5, 121.2]	0.81
**Type of HF**				0.037
HFrEF	181 (49.5)	127 (45.7)	54 (61.4)	
HFmrEF	84 (23.0)	68 (24.5)	16 (18.2)	
HFpEF	101 (27.6)	83 (29.9)	18 (20.5)	
**Ischemic cardiomyopathy**	124 (33.9)	90 (32.4)	34 (38.6)	0.34
**LVEF, %**	45.0 [32.8, 55.0]	45.0 [33.2, 55.0]	39.0 [30.0, 50.0]	0.069
**Medical history/ Comorbidities**				
**Myocardial infarction**	145 (39.6)	107 (38.5)	38 (43.2)	0.51
**Atrial fibrillation**	245 (66.9)	191 (68.7)	54 (61.4)	0.25
**Hypertension**	271 (74.0)	212 (76.3)	59 (67.0)	0.11
**Diabetes mellitus**	170 (46.4)	124 (44.6)	46 (52.3)	0.26
**Previous stroke/TIA**	80 (21.9)	64 (23.0)	16 (18.2)	0.42
**COPD**	91 (24.9)	73 (26.3)	18 (20.5)	0.34
**OSAS**	69 (18.9)	47 (16.9)	22 (25.0)	0.12
**Chronic kidney disease** ^**†**^	264 (72.1)	193 (69.4)	71 (80.7)	0.055
**Current or former smoker**	183 (50.0)	139 (50.0)	44 (50.0)	1.00
**Heart failure related medical or device therapy**				
**RAAS inhibitor**	219 (59.8)	168 (60.4)	51 (58.0)	0.77
**ACE inhibitor**	111 (30.3)	82 (29.5)	29 (33.0)	0.63
**ARB**	60 (16.4)	50 (18.0)	10 (11.4)	0.20
**ARNI**	48 (13.1)	36 (12.9)	12 (13.6)	1.00
**Beta-blocker**	302 (82.5)	228 (82.0)	74 (84.1)	0.78
**MRA**	223 (60.9)	170 (61.2)	53 (60.2)	0.98
**Loop diuretic**	350 (95.6)	264 (95.0)	86 (97.7)	0.42
**Thiazide diuretic**	8 (2.2)	5 (1.8)	3 (3.4)	0.63
**SGLT2 inhibitor**	21 (5.7)	16 (5.8)	5 (5.7)	1.00
**Statin**	239 (65.3)	170 (61.2)	69 (78.4)	0.005
**CRT**	56 (15.3)	38 (13.7)	18 (20.5)	0.17
**ICD**	68 (18.6)	52 (18.7)	16 (18.2)	1.00
**Characteristics at time of first outpatient treatment**				
**Heart rate, beats per minute**	76.5 (15.2)	75.2 (13.4)	80.6 (19.1)	0.004
*Missing*	*3 (0.82%)*	*3 (1.08%)*	*0 (0%)*	
**SBP, mmHg**	123.3 (20.8)	123.9 (20.2)	121.2 (22.5)	0.29
**DBP, mmHg**	67.7 (12.6)	67.4 (12.3)	68.5 (13.4)	0.47
Home dosage of furosemide equivalent, mg/day*	80.0 [40.0, 160.0]	80.0 [40.0, 120.0]	80.0 [40.0, 160.0]	0.014
**Home dosage of loop diuretic**				0.022
Low, 0–80 mg furosemide or equivalent	230 (62.8)	183 (65.8)	47 (53.4)	
Middle, 81–160 mg furosemide or equivalent	84 (23.0)	63 (22.7)	21 (23.9)	
High, > 160 mg furosemide or equivalent	52 (14.2)	32 (11.5)	20 (22.7)	
**Laboratory values at time of first outpatient treatment**				
**Haemoglobin, mmol/L**	7.7 (1.2)	7.8 (1.2)	7.3 (1.2)	0.003
*Missing*	*2 (0.55%)*	*2 (0.72%)*	*0 (0%)*	
**Sodium, mmol/L**	138.6 (3.8)	138.8 (3.6)	138.0 (4.2)	0.083
**Potassium, mmol/L**	4.3 (0.6)	4.3 (0.5)	4.3 (0.6)	0.41
**Urea, mmol/L**	11.9 [8.8, 17.0]	11.0 [8.4, 16.2]	14.0 [9.5, 18.6]	0.004
*Missing*	*1 (0.27%)*	*1 (0.36%)*	*0 (0%)*	
**Serum creatinine, umol/L**	129.0 [96.2, 169.8]	123.5 [92.2, 163.0]	148.0 [110.8, 201.5]	<0.001
**NT-proBNP, pmol/L**	437.5 [168.0, 950.5]	344.5 [152.5, 730.5]	814.0 [286.5, NA]	<0.001
**eGFR, ml/min/1.73m** ^**2**^	44.5 (19.9)	46.4 (19.7)	38.3 (19.3)	0.001

*Variables are presented as mean (SD), median [IQR] or number (%).*

*ACE, angiotensin-converting enzyme; ARB, angiotensin-receptor blocker; ARNI, angiotensin receptor-neprilysin inhibitor; BMI, Body Mass Index; COPD, chronic obstructive pulmonary disease; CRT, cardiac resynchronization therapy; DBP, diastolic blood pressure; eGFR, estimated glomerular filtration rate; HF, heart failure; HFH; heart failure related (re-)hospitalization; HFrEF, Heart failure with reduced ejection fraction; HFmrEF, heart failure with mildly reduced ejection fraction; HFpEF, heart failure with preserved ejection fraction; ICD, implantable cardioverter-defibrillator; LVEF, left ventricular ejection fraction; MRA, mineralocorticoid receptor antagonist; NT-proBNP, N-terminal pro-B-type natriuretic peptide; OSAS, obstructive sleep apnea syndrome; RAAS, renin–angiotensin–aldosterone system; SBP, systolic blood pressure; SGLT2, sodium-glucose co-transporter 2; TIA, transient ischemic attack.*

*† Chronic kidney disease is defined as an eGFR < 60 mL/min/1.73m*^*2*^ *for at least 3 months.*

*40 mg furosemide is equivalent to 1 mg bumetanide.

**Table 3:** Diuretic use at outpatient treatment in hospital A (n = 366).

	Overall n = 366	No HFH n = 278	HFH n = 88
**Bolus received, *n (%)***	364 (99.5)	277 (99.6)	87 (98.9)
**Dosage of bolus diuretic in mg, *mean (SD)***	117 (36)	116 (31)	121 (47)
**Perfusor received, *n (%)***	172 (47.3)	126 (45.7)	46 (52.3)
**Dosage of perfusor diuretic in mg, *mean (SD)***	196 (59)	196 (57)	197 (66)
**Total diuretic dosage in mg, *mean (SD)***	210 (110)	205 (110)	223 (111)
**Repeated lounge treatments within 1 week, *n (%)***			
**1 repeated treatment**	94 (25.7)	70 (25.2)	24 (27.3)
**> 1 repeated treatment**	51 (13.9)	40 (14.4)	11 (12.5)

*No HFH, patients without a heart failure related (re-)hospitalization within 30 days; HFH, patients with a heart failure related (re-)hospitalization within 30 days.*

*All p-values were not significant (p > 0.05).*

*n = 3 missing (all in no HFH group); **n = 2 missing (all in no HFH group); *** n = 4 missing (all in no HFH group).

Patients who reached the primary endpoint had a lower mean age than those who remained free of the endpoint. Furthermore, they had more frequently experienced HF-related hospitalizations in the year before the index outpatient treatment, had a significantly higher heart rate, albeit within the normal range, higher NT-proBNP levels, and a worse kidney function than those without a HF (re-)hospitalization (Table 2). Besides more frequently higher dosage of loop diuretics, the other baseline characteristics and therapy rates were comparable between both groups.

The outpatient treatment required multiple sessions with IV diuretics for decongestion in 145 patients (40%): 94 patients had one additional IV diuretic treatment within a week and 51 patients had more than one additional treatments within a week. The number of patients requiring multiple sessions did not differ significantly between patients with and without the primary outcome (Table 3).

### Primary endpoint

Multivariable regression identified five predictors of 30-day HF related (re-)hospitalization. Internal validation showed that a shrinkage factor of 0.919 was needed to correct the regression coefficients for optimism. After correction, doubling of NT-proBNP was a significant predictor of the primary endpoint (OR 1.34, 95% CI 1.14–1.57). Furthermore, a higher age, higher eGFR, and higher concentrations of sodium and haemoglobin were predictive of a lower risk of 30-day HF (re-)hospitalizations, with ORs < 1 (Table 4).

**Table 4:** Predictors of 30-day HF (re-)hospitalization in hospital A (n = 366).

	Beta*	OR	95% CI	P-value
**Intercept**	10.28			
**Age, years**	−0.05	0.96	(0.93 - 0.98)	<0.001
**eGFR (CKD-EPI formula), mL/min/1.73m** ^**2**^	−0.15	0.86	(0.74 - 1.01)	0.063
**Log2(NT-proBNP), pmol/L**	0.29	1.34	(1.14 - 1.57)	<0.001
**Sodium, mmol/L**	−0.06	0.94	(0.88 - 1.01)	0.085
**Haemoglobin, mmol/L**	−0.25	0.78	(0.62 - 0.98)	0.031

*CI, confidence interval; CKD-EPI, Chronic Kidney Disease Epidemiology Collaboration; eGFR, estimated glomerular filtration rate; NT-proBNP, N-terminal pro-B-type natriuretic peptide; OR, odds ratio.*

*Estimates presented are shrunken after internal validation with shrinkage factor 0.919.

*ORs are calculated per 1-unit increase for age, sodium, and haemoglobin; per 10-unit increase for eGFR; and per doubling for NT-proBNP.*

#### Performance and exploratory validation.

**Fig 1:**
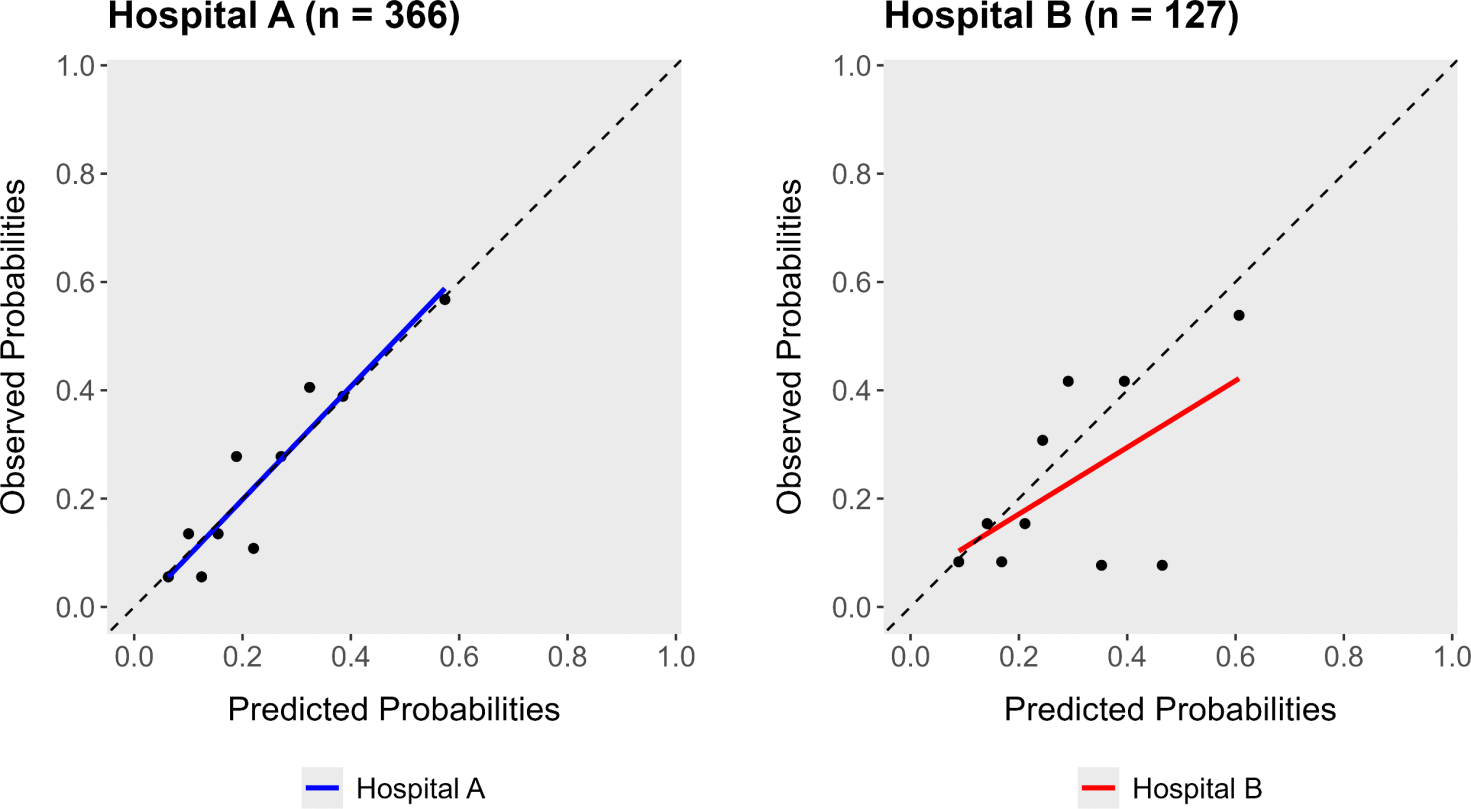
Calibration plots. Calibration plots of predicted probabilities versus observed probabilities of 30-day HF (re-)hospitalization in hospital A (left plot) and hospital B (right plot). HF; heart failure.

In hospital A, the adjusted predictors showed acceptable discrimination with AUC of 0.73 and good calibration (Table 5 and Fig 1A).

**Table 5:** Performance in hospital A (n = 366) and hospital B (n = 127).

	Apparent model	Internal validation model	External validation model
**AUC**	0.73	0.73	0.65
**Nagelkerke R** ^**2**^	0.19	0.19	0.06
**Calibration intercept**	0.00	0.09	−0.53
**Calibration slope**	1.00	1.09	0.81

*AUC, area under the curve. Apparent and internal validation model coefficients were derived in hospital A and applied without re-estimation in hospital B for exploratory purposes (external validation model).*

An exploratory validation of these predictors was performed in 127 patients from hospital B, with a similar 30-day HF (re-)hospitalization rate to hospital A (n = 29, 22.8%, p = 0.804). The patient characteristics of hospital A versus hospital B are outlined in S4 Table in S1 File. Applying the identified predictors from hospital A to the patients from hospital B led to decreased calibration and discrimination as shown in Fig 1B and Table 5.

### Secondary endpoints

#### Heart failure-related hospitalizations within six months.

A total of 142 (39%) patients of hospital A experienced an HF-related (re-)hospitalization within six months. Doubling of NT-proBNP, history of a HF hospitalization in the previous 12 months, and higher home loop diuretic dosages were associated with a higher risk of HF-related (re-)hospitalization in six months. A higher age, higher eGFR, and higher concentrations of haemoglobin were associated with a lower risk of HF (re-)hospitalization in six months. Predictors and corresponding ORs are presented in Table 6.

#### Mortality.

A total of 57 (16%) patients of hospital A died within six months. A higher age, higher home loop diuretic dosages, and doubling of NT-proBNP were predictors for higher risk of mortality within six months, whereas higher concentrations of sodium were associated with a lower risk (see Table 6).

**Table 6:** Predictors associated with 6-month HF (re-)hospitalization and mortality in hospital A (n = 366).

	6-month HF (re-)hospitalization			6-month mortality		
	OR	95% CI	P-value	OR	95% CI	P-value
Age, years	0.97	(0.95 - 1.00)	0.021	1.04	(1.00 - 1.07)	0.041
History of HFH < 12 months	1.58	(0.89 - 2.86)	0.12			
Home dosage of loop diuretic: low 0–80 mg	Ref	–	–	Ref	–	–
Home dosage of loop diuretic: middle 81–160 mg	1.56	(0.89 - 2.73)	0.12	1.88	(0.88 - 3.96)	0.096
Home dosage of loop diuretic: high > 161 mg	2.45	(1.25 - 4.87)	0.009	2.08	(0.81 - 5.07)	0.12
eGFR, ml/min/1.73m^2^	0.98	(0.97 - 1.00)	0.018			
log2(NT-proBNP)	1.33	(1.16 - 1.54)	<0.001	1.80	(1.47 - 2.25)	<0.001
Haemoglobin, mmol/L	0.85	(0.69 - 1.03)	0.10			
Sodium, mmol/L				0.90	(0.84 - 0.97)	0.008

*CI, confidence interval; eGFR, estimated glomerular filtration rate; HF, heart failure; HFH, heart failure related hospitalization; NT-proBNP, N-terminal pro-B-type natriuretic peptide; OR, odds ratio.*

*ORs are calculated per 1-unit increase for age, sodium, and haemoglobin; per 10-unit increase for eGFR; and per doubling for NT-proBNP.*

#### Repeated outpatient treatment.

A total of 113 (31%) patients of hospital A underwent repeated outpatient treatment with IV diuretics. Only eGFR remained as predictor after applying the stepwise backward procedure (OR per 10-unit increase: 0.91, 95% CI 0.81–1.02, p-value = 0.113).

### Sensitivity analysis

Sensitivity analysis for the primary endpoint in hospital A for the three different types of HF (HFrEF, HFmrEF, and HFpEF) yielded consistent results across subgroups, except that NT-proBNP showed no association in the HFpEF group (S5 Table in S1 File).

## Discussion

**Fig 2:**
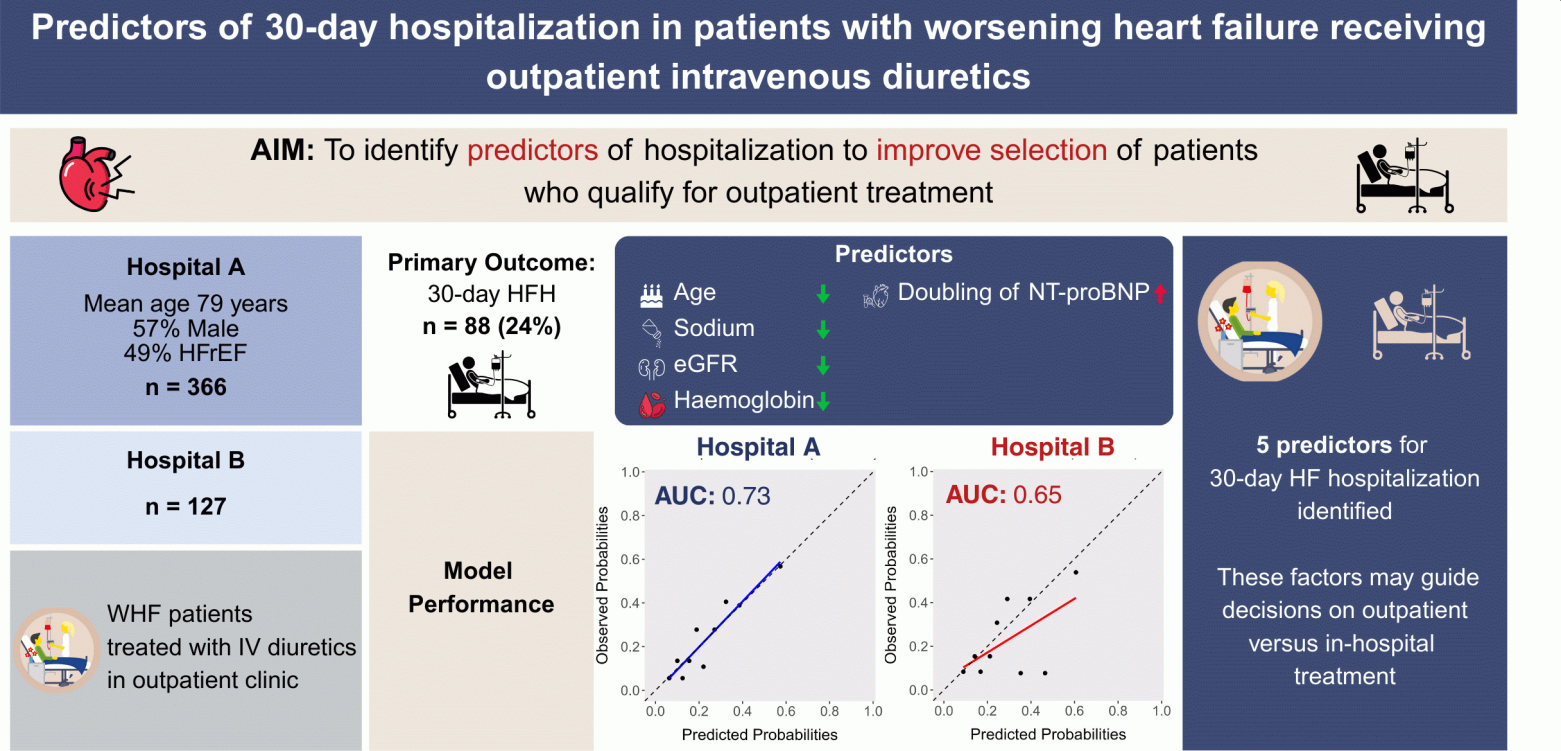
Graphical abstract. Predictors of 30-day hospitalization in patients with worsening heart failure receiving outpatient intravenous diuretics. AUC, area under the curve; eGFR, estimated glomerular filtration rate; HFH, heart failure hospitalization; HFrEF, heart failure with reduced ejection fraction; IV, intravenous; NT-proBNP, N-terminal pro-B-type natriuretic peptide; WHF, worsening heart failure.

This study addressed the practical issue of identifying which WHF patients may benefit from outpatient treatment with IV diuretics, and which may be better suited for in-hospital treatment. Our main findings indicated that higher baseline NT-proBNP levels, as well as lower age, eGFR, sodium, and haemoglobin were predictors of 30-day HF hospitalization (HFH) (Fig 2). Furthermore, baseline NT-proBNP and oral loop diuretic dosage were strong predictors of mortality and HFH at 6 months.

In our cohort – one of the largest outpatient IV diuretic treatment cohorts to date – we observed a 30-day HFH rate of 24%. Although a considerable number of WHF patients was still hospitalized within 30 days after outpatient treatment, in the majority of patients a HFH could be successfully averted, confirming the valuable contribution of outpatient treatment in HF healthcare.

Our findings support the current practical guideline that recommends against outpatient treatment in patients with severely impaired renal function and/or electrolyte disturbances [7]. In addition, our findings indicate that patients presenting with anemia or markedly elevated NT-proBNP levels may also be better suited for direct in-hospital treatment instead of outpatient IV diuretic treatment.

Current evidence on prognostic factors to identify ‘high risk’ patients for outpatient treatment is limited. For instance, Ryder et al identified three independent predictors in 107 patients: lower systolic blood pressure, oral diuretic increase, and beta-blocker use. They did not clearly define which variables were included in the multivariable model but stated that variables were selected based on being theoretically reasonable and significant univariable associations [9]. Similarly, our previous study evaluated a predefined set of seven potential predictors in a smaller subset of our current cohort [3]. The current study expands this work by using a more comprehensive predictor identification process, with a larger cohort and validation in an independent hospital dataset.

Our findings in this cohort of WHF patients align with prior research that identified higher NT-proBNP, and lower eGFR, sodium, and haemoglobin levels as predictors of HFH in different HF populations, such as the aforementioned ‘BCN Bio-HF Calculator’ [10]. Moreover, the association of higher oral loop diuretic dosages – likely reflecting more severe congestion and/or diuretic resistance – with 6-month hospitalization and mortality is consistent with previous studies [7,10,14].

Interestingly, we found that increasing age was predictive of a lower 30-day HFH risk, whereas this was also associated with a higher mortality risk. A possible explanation of this protective effect of age on hospitalization risk can be sought in survivorship bias: older, frailer patients may be more likely to be referred directly for hospitalization, leaving a relatively healthier subset of elder patients for outpatient treatment [15].

The predictors of 30-day HFH remained constant in sensitivity analyses except NT-proBNP for HFpEF patients. This finding contrasts with Pocock et al., who demonstrated NT-proBNP as a powerful predictor of HFH and cardiovascular mortality in two HFpEF cohorts [16]. The absence of this association in our cohort may be explained by the lower NT-proBNP levels in HFpEF compared to HFrEF and HFmrEF patients, combined with fewer primary endpoint events in the HFpEF patients (S6 Table in S1 File). Additionally, the higher proportion of females and higher BMI among HFpEF patients in our study may have contributed to lower NT-proBNP levels [17]. Moreover, a meta-analysis of MRA treatment trials reported that HFpEF itself is associated with lower hospitalization rates compared to HFrEF, which could partially explain our findings [18].

Although our final set of predictors yielded acceptable discrimination and calibration in hospital A, performance decreased when applied to hospital B. Possible explanations for the reduced performance in hospital B can be found in differences in baseline characteristics or subtle differences in procedures despite following the same outpatient treatment protocol. This study serves as an important first step towards developing a valid prediction model to address the unmet need for improved patient selection for outpatient treatment with IV diuretics versus direct in-hospital treatment.

### Strengths and limitations

Strengths of this study include the use of prognostic variables that are readily available in routine clinical practice, supported by the minimal rate of missing values in this retrospective cohort. The study cohort is also representative of routine HF management in the Netherlands, with a relative high uptake of guideline directed medical therapy (GDMT), diverse heart failure phenotypes, an older average age, and a considerable burden of comorbidities, rather than a selected trial population. Furthermore – as previously stated – this study includes one of the largest cohorts of patients treated with IV diuretics in an outpatient setting, offering valuable insights into this treatment approach. Generalizability is further supported by the relative high proportion of patients with a language barrier (22%). This implicates a mixed population, unlike the predominantly Caucasian cohorts seen in similar studies [11]. However, data on race or ethnic background to support this implication were not collected.

Despite these strengths, several study limitations need to be considered. The hospital B cohort was too small to have enough events for a proper external validation. The limited sample size limits the interpretation of accuracy metrics of the set of predictors. The AUC of 0.73 of the internal validation is acceptable but still moderate, suggesting that important predictors may be overlooked. For example, comorbidities were not included in the model, but no significant differences were observed in comorbidities between the endpoint groups (Table 2). Additionally, implementation of GDMT such as CRT and RAAS-inhibitors were included in our model, though these are not applicable for HFpEF patients. Vital parameters at time of outpatient treatment were not included in our analysis, as our aim was to predict risk of hospitalization *prior* to arrival at the outpatient clinic. These parameters are typically only available once patients have already presented for care. However, we acknowledge that including vital parameters could enhance predictive performance and clinical applicability in certain settings. In addition, our current data did not include objective measures of congestion severity such as ultrasound findings or urine sodium. Lastly, by including readily available indicators, we excluded biomarkers such as high-sensitive cardiac troponin T and ST2, which have shown predictive ability in previous research [10,16].

### Implications for future research and clinical practice

To advance toward use in routine patient management, future studies should not only externally validate our identified predictors but also identify additional predictors of successful outpatient treatment with IV diuretics. Future research on outpatient IV diuretic treatment should also focus on markers for treatment response, such as urine output, urine sodium levels, lung ultrasound, and NT-proBNP follow-up and echocardiographic assessment of filling pressures.

Additionally, we observed a higher prevalence of language barriers in patients with a HFH compared to those without a rehospitalization in hospital A (27.3 vs 20.5%, p = 0.24). This higher rehospitalization rate may reflect different patient- and physician-related factors variables, including lower medication adherence due to communication problems and/or different cultural backgrounds and health beliefs, and physician preferences or hospitalization when communication is challenging. Presence of a language barrier might also be associated with lower health literacy and lower socioeconomic status (SES), which in itself is associated with higher risk of HF-related hospitalizations [19]. For future research it would be interesting to collect more data on patients SES and include this in risk predictions.

Finally, reducing heterogeneity between outpatient treatment centers – including differences in treatment protocol and patient selection – would strengthen the evidence base for outpatient IV diuretic treatment and improve generalizability of a future risk prediction tool. A critical step forward will be the design of a multicenter randomized controlled trial (RCT) to compare outpatient IV diuretic treatment versus standard in-hospital care for WHF patients.

## Conclusion

In patients with WHF receiving outpatient day-care IV diuretic treatment, age, eGFR, NT-proBNP, sodium and haemoglobin predicted 30-day HF hospitalization. Despite the moderate performance in external data, these routinely available clinical parameters can help guide clinical decision-making on day-care outpatient versus ‘classic’ in-hospital treatment.

## Supporting information

S1 File (PDF)
