# Supplementary material for: Predictors of 30-day hospitalization in patients with worsening heart failure receiving outpatient intravenous diuretics
Source: PLoS One. 2026 Feb 17;21(2):e0342263. doi: 10.1371/journal.pone.0342263 (PMC12912686; doi:10.1371/journal.pone.0342263)
Supplement: S1 File — (PDF) [file pone.0342263.s001.pdf]

Predictors of 30-day hospitalization in patients with worsening heart failure receiving outpatient intravenous diuretics

Willemijn A. van Maarschalkerwaart, MD[1]; Eric Wierda, MD, PhD[2]; Dominique de Boer[2]; Nini H. Jonkman[3]; Eric Boersma, MSc, PhD[4]; Jasper J. Brugts, MD, PhD[4]; Loek van Heerebeek, MD, PhD[1]

SUPPORTING INFORMATION

Table of contents

S1 Fig. Flowchart of patient inclusion 3

S1 Table. Predictor variables 4

S2 Table. Variance inflation factors 5

S3 Table. Spearman correlations 5

S4 Table. Baseline characteristics of total population, stratified by hospital A (n=366) and hospital B (n=127) 6

S5 Table. Sensitivity analysis of predictors of 30-day HF (re-)hospitalization in hospital A (n=366 patients), stratified by heart failure type 8

S6 Table. Baseline characteristics of hospital A (n = 366), stratified by type of heart failure 9

S1 Fig. Flowchart of patient inclusion

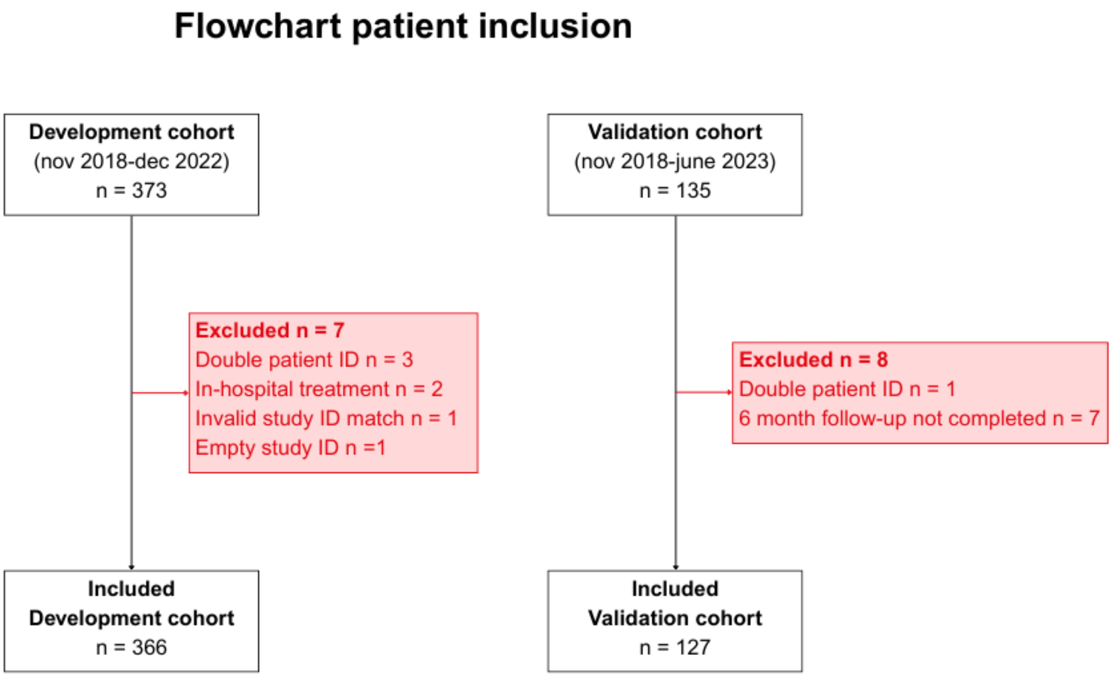

S1 Table. Predictor variables

Predictor variable	Definition	Coding
Age	Age at time of first outpatient treatment	Continuous, years
Sex	Male or female	Binary, male/female
BMI	Body mass index calculated from length and weight at time of first outpatient treatment	Continuous, kg/m^2
Previous HF hospitalization	Number of heart failure related hospitalizations in the 12 months prior to time of first outpatient treatment	Continuous, number of hospitalizations
Dosage oral loop diuretics	Home dosage of furosemide or furosemide equivalent with 1 mg bumetanide being equivalent to 40mg furosemide, defined as low (0-80 mg furosemide or equivalent), middle (81-160 mg furosemide or equivalent) or high (>160 mg furosemide or equivalent)	Categorical, Low, Middle and High (> 160mg)
Type of HF	Type of heart failure based on primary heart failure diagnosis, defined as HFrEF (EF <40%), HFmrEF (EF 40-49%) or HFpEF (EF ≥50%)	Categorical, HFrEF, HFmrEF and HFpEF
Kidney function	Kidney function based on eGFR (CKD-EPI formula) at time of first outpatient treatment	Continuous, $ml/min/1.73m^2$
NT-proBNP	NT-proBNP level at time of first outpatient treatment	Continuous, pmol/L
Sodium	Sodium level at time of first outpatient treatment	Continuous, mmol/L
Haemoglobin	Haemoglobin level at time of first outpatient treatment	Continuous, mmol/L
CRT-D/CRT-P	Presence of either CRT-D or CRT-P device at time of first outpatient treatment	Binary, yes/no
ACE/ARB/ARNI	Use of either ACE inhibitor, ARB or ARNI at time of first outpatient treatment	Binary, yes/no
Language barrier	Presence of a language barrier at time of first outpatient treatment (based on documentation in EPD: 'language barrier' or need of an official interpreter or family member to translate)	Binary, yes/no

ACE, angiotensin-converting enzyme; ARB, angiotensin-receptor blocker; ARNI, angiotensin receptor-neprilysin inhibitor; BMI, Body Mass Index; CRT-D, cardiac resynchronization therapy with a defibrillator; CRT-P, cardiac resynchronization therapy with a pacemaker; eGFR, estimated glomerular filtration rate; HF, heart failure; HFrEF, Heart failure with reduced ejection fraction; HFmrEF, heart failure with mildly reduced ejection fraction; HFpEF, heart failure with preserved ejection fraction; NT-proBNP, N-terminal pro-B-type natriuretic peptide.

S2 Table. Variance inflation factors

Predictor	VIF
Age	1.14
Sex	1.10
BMI	1.15
History of HFH <12 months	1.06
Home dosage of loop diuretic	1.06
Type HF	1.11
eGFR	1.19
log2(NT-proBNP)	1.23
Sodium	1.06
Haemoglobin	1.09
CRT	1.06
RAAS inhibitor	1.07
Language barrier	1.10

VIF, variance inflation factor.
*Presented VIF values are Generalized VIF values calculated as GVIF^(1/(2*Df)).*
VIF > 5 considered indicative of problematic multicollinearity.

S3 Table. Spearman correlations

Spearman correlations	Age	BMI	History of HFH <12 months	eGFR	log2(NT-proBNP)	Sodium	Haemoglobin
**Age**	1	-0,28	0,01	-0,24	0,16	0,01	0
**BMI**	-0,28	1	0,11	0,01	-0,33	0,09	0,01
**History of HFH <12 months**	0,01	0,11	1	-0,27	0,1	-0,02	-0,14
**eGFR**	-0,24	0,01	-0,27	1	-0,39	-0,04	0,2
**log2(NT-proBNP)**	0,16	-0,33	0,1	-0,39	1	0,01	-0,03
**Sodium**	0,01	0,09	-0,02	-0,04	0,01	1	0,04
**Haemoglobin**	0	0,01	-0,14	0,2	-0,03	0,04	1

BMI, body mass index; HFH, heart failure hospitalization; eGFR, estimated glomerular filtration rate; NT-proBNP, N-terminal pro-B-type natriuretic peptide.

S4 Table. Baseline characteristics of total population, stratified by hospital A (n=366) and hospital B (n=127)

	Hospital A<br>n = 366	Hospital B<br>n = 127	P-value
**Outcome 30-day HF (re-)hospitalization**	88 (24.0)	29 (22.8)	0.88
**Age, years**	76.1 (10.1)	75.4 (9.2)	0.48
**Male**	207 (56.6)	83 (65.4)	0.10
**BMI, kg/m^2**	29.7 (6.8)	26.8 (5.1)	<0.001
**Language barrier**	81 (22.1)	0 (0.0)	<0.001
**HF hospitalization within previous 12 months**	270 (73.8)	85 (66.9)	0.17
**Number of HF hospitalizations in previous year**	2.0 [1.0, 2.0]	2.0 [1.0, 2.0]	0.14
**HF duration in months**	47.5 [16.0, 116.5]	48.0 [13.0, 118.5]	0.72
**Type of HF**			0.090
HFrEF	181 (49.5)	74 (58.3)	
HFmrEF	84 (23.0)	30 (23.6)	
HFpEF	101 (27.6)	23 (18.1)	
**Ischemic cardiomyopathy**	124 (33.9)	43 (33.9)	1.00
**LVEF, %**	45.0 [32.8, 55.0]	35.0 [28.0, 45.0]	<0.001
**Medical history / Comorbidities**			
**Myocardial infarction**	145 (39.6)	51 (40.2)	1.00
**Atrial fibrillation**	245 (66.9)	81 (63.8)	0.59
**Hypertension**	271 (74.0)	64 (50.4)	<0.001
**Diabetes mellitus**			0.036
**No**	196 (53.6)	80 (63.0)	
Diabetes mellitus type 1	0 (0.0)	1 (0.8)	
Diabetes mellitus type 2	170 (46.4)	46 (36.2)	
**Previous stroke/TIA**	80 (21.9)	19 (15.0)	0.12
**COPD**	91 (24.9)	25 (19.7)	0.29
**OSAS**	69 (18.9)	16 (12.6)	0.14
**Chronic kidney disease**	264 (72.1)	107 (84.3)	0.009
**Heart failure related medical or device therapy**			
**RAAS inhibitor**	219 (59.8)	69 (54.3)	0.33
**ACE inhibitor**	111 (30.3)	26 (20.5)	0.043
**ARB**	60 (16.4)	15 (11.8)	0.27
**ARNI**	48 (13.1)	28 (22.0)	0.024
**Beta-blocker**	302 (82.5)	105 (82.7)	1.00
**MRA**	223 (60.9)	75 (59.1)	0.79
**Loop diuretic**	350 (95.6)	125 (98.4)	0.24
**Thiazide diuretic**	8 (2.2)	10 (7.9)	0.008
**SGLT2 inhibitor**	21 (5.7)	21 (16.5)	<0.001
**Statin**	239 (65.3)	65 (51.2)	0.007
**CRT**	56 (15.3)	21 (16.5)	0.85
**ICD**	68 (18.6)	34 (26.8)	0.066

**Characteristics at time of first outpatient treatment**			
**Heart rate, beats per minute**	76.5 (15.2)	75.3 (14.4)	0.44
Missing	*3 (0.82%)*	*0 (0%)*	
**SBP, mmHg**	123.3 (20.8)	119.8 (20.8)	0.11
**DBP, mmHg**	67.7 (12.6)	69.6 (12.2)	0.14
**Home dosage of furosemide equivalent, mg/day***	80.0 [40.0, 160.0]	160.0 [80.0, 200.0]	<0.001
**Home dosage of loop diuretic**			<0.001
Low (0-80 mg furosemide or equivalent	230 (62.8)	41 (32.3)	
Middle (81-160 mg furosemide or equivalent	84 (23.0)	30 (23.6)	
High, >160 mg furosemide or equivalent	52 (14.2)	56 (44.1)	
**Laboratory values at time of first outpatient treatment**			
**Haemoglobin, mmol/L**	7.7 (1.2)	7.7 (1.2)	0.85
Missing	*2 (0.55%)*	*0 (0%)*	
**Sodium, mmol/L**	138.6 (3.8)	138.3 (3.6)	0.37
**Potassium, mmol/L**	4.3 (0.6)	4.3 (0.6)	0.80
**Urea, mmol/L**	11.9 [8.8, 17.0]	15.0 [9.6, 21.4]	<0.001
Missing	*1 (0.27%)*	*1 (0.79%)*	
**Serum creatinine, umol/L**	129.0 [96.2, 169.8]	156.0 [112.5, 187.5]	<0.001
**NT-proBNP, pmol/L**	437.5 [168.0, 950.5]	653.0 [280.2, NA]	0.001
**eGFR, ml/min/1.73m^2**	44.5 (19.9)	39.6 (20.2)	0.017
Missing	*0 (0%)*	*1 (0.79%)*	

Variables are presented as mean (SD), median [IQR] or number (%).
ACE, angiotensin-converting enzyme; ARB, angiotensin-receptor blocker; ARNI, angiotensin receptor-neprilysin inhibitor; BMI, Body Mass Index; COPD, chronic obstructive pulmonary disease; CRT, cardiac resynchronization therapy; DBP, diastolic blood pressure; eGFR, estimated glomerular filtration rate; HF, heart failure; HFrEF, Heart failure with reduced ejection fraction; HFmrEF, heart failure with mildly reduced ejection fraction; HFpEF, heart failure with preserved ejection fraction; ICD, implantable cardioverter-defibrillator; LVEF, left ventricular ejection fraction; MRA, mineralocorticoid receptor antagonist; NT-proBNP, N-terminal pro-B-type natriuretic peptide; OSAS, obstructive sleep apnoea syndrome; RAAS, renin–angiotensin–aldosterone system; SBP, systolic blood pressure; SGLT2, sodium-glucose co-transporter 2; TIA, transient ischemic attack.
**40mg furosemide is equivalent to 1mg bumetanide.*

S5 Table. Sensitivity analysis of predictors of 30-day HF (re-)hospitalization in hospital A (n=366 patients), stratified by heart failure type

	HFrEF (n = 181)			HFmrEF (n = 84)			HFpEF (n = 101)			Total development cohort (n = 366)		
	**Beta**	**OR**	**95% CI**	**Beta**	**OR**	**95% CI**	**Beta**	**OR**	**95% CI**	**Beta**	**OR**	**95% CI**
**Age, years**	-0.04	0.97	(0.93 - 1.00)	-0.05	0.95	(0.87 - 1.03)	-0.06	0.94	(0.88 - 1.00)	-0.04	0.96	(0.93 - 0.98)
**eGFR, ml/min/1.73m^2**	-0.07	0.93	(0.76 - 1.14)	-0.26	0.77	(0.51 - 1.10)	-0.31	0.73	(0.49 - 1.02)	-0.15	0.86	(0.74 - 1.01)
**log2(NT-proBNP), pmol/L**	0.44	1.55	(1.23 - 2.00)	0.57	1.76	(1.06 - 3.18)	-0.02	0.98	(0.71 - 1.37)	0.29	1.34	(1.14 - 1.57)
**Sodium, mmol/L**	-0.06	0.94	(0.86 - 1.03)	-0.09	0.92	(0.79 - 1.06)	-0.04	0.96	(0.82 - 1.12)	-0.06	0.94	(0.88 - 1.01)
**Haemoglobin, mmol/L**	-0.22	0.80	(0.59 - 1.07)	-0.54	0.58	(0.32 - 1.00)	-0.31	0.74	(0.42 - 1.26)	-0.25	0.78	(0.62 - 0.98)

CI, confidence interval; eGFR, estimated glomerular filtration rate; HFmrEF, heart failure with mildly reduced ejection fraction; HFpEF, heart failure with preserved ejection fraction; HFrEF, heart failure with reduced ejection fraction; NT-proBNP, N-terminal pro-B-type natriuretic peptide; OR, odds ratio.

ORs are calculated per 1-unit increase for age, sodium, and haemoglobin; per 10-unit increase for eGFR; and per doubling for NT-proBNP.

S6 Table. Baseline characteristics of hospital A (n = 366), stratified by type of heart failure

	HFrEF<br>n = 181	HFmrEF<br>n = 84	HFpEF<br>n = 101	P-value
**Outcome 30-day HF (re-)hospitalization**	73 (28.6)	22 (19.3)	22 (17.7)	0.03
**Age, years**	73.6 (10.8)	79.3 (8.2)	77.8 (8.9)	<0.001
**Male**	128 (70.7)	44 (52.4)	35 (34.7)	<0.001
**BMI, kg/m2**	28.5 (5.7)	30.2 (7.9)	31.3 (7.2)	0.002
**Language barrier**	35 (19.3)	20 (23.8)	26 (25.7)	0.42
**HF hospitalization within previous 12 months**	126 (69.6)	64 (76.2)	80 (79.2)	0.18
**Number of HF hospitalizations in previous 12 months**	2.0 [1.0, 2.0]	2.0 [2.0, 2.0]	2.0 [2.0, 2.0]	0.18
**HF duration in months**	66.0 [21.0, 154.0]	44.0 [11.8, 103.2]	29.0 [13.0, 75.0]	0.002
**Ischemic cardiomyopathy**	88 (48.6)	28 (33.3)	8 (7.9)	<0.001
**LVEF, %**	32.0 [25.0, 36.0]	45.0 [45.0, 50.0]	55.0 [55.0, 60.0]	<0.001
**Medical history / Comorbidities**				
**Myocardial infarction**	93 (51.4)	32 (38.1)	20 (19.8)	<0.001
**Atrial fibrillation**	110 (60.8)	56 (66.7)	79 (78.2)	0.01
**Hypertension**	127 (70.2)	63 (75.0)	81 (80.2)	0.18
**Diabetes mellitus**	79 (43.6)	42 (50.0)	49 (48.5)	0.56
**Previous stroke/TIA**	41 (22.7)	17 (20.2)	22 (21.8)	0.91
**COPD**	40 (22.1)	24 (28.6)	27 (26.7)	0.46
**OSAS**	27 (14.9)	18 (21.4)	24 (23.8)	0.15
**Chronic kidney disease**	125 (69.1)	66 (78.6)	73 (72.3)	0.28
**Current or former smoker**	90 (49.7)	46 (54.8)	47 (46.5)	0.54
**Heart failure related medical or device therapy**				
**RAAS inhibitor**	121 (66.9)	42 (50.0)	56 (55.4)	0.02
**ACE inhibitor**	60 (33.1)	22 (26.2)	29 (28.7)	0.47
**ARB**	23 (12.7)	12 (14.3)	25 (24.8)	0.03
**ARNI**	38 (21.0)	8 (9.5)	2 (2.0)	<0.001
**Beta-blocker**	157 (86.7)	69 (82.1)	76 (75.2)	0.05
**MRA**	122 (67.4)	43 (51.2)	58 (57.4)	0.03
**Loop diuretic**	173 (95.6)	81 (96.4)	96 (95.0)	0.90
**Thiazide diuretic**	5 (2.8)	2 (2.4)	1 (1.0)	0.61
**SGLT2 inhibitor**	12 (6.6)	3 (3.6)	6 (5.9)	0.61
**Statin**	127 (70.2)	52 (61.9)	60 (59.4)	0.14
**CRT**	44 (24.3)	10 (11.9)	2 (2.0)	<0.001
**ICD**	58 (32.0)	7 (8.3)	3 (3.0)	<0.001
**Characteristics at time of first outpatient treatment**				
**Heart rate, beats per minute**	78.2 (15.7)	74.9 (15.7)	75.0 (13.6)	0.13
**SBP, mmHg**	118.5 (20.8)	126.1 (20.5)	129.6 (18.9)	<0.001
**DBP, mmHg**	69.3 (13.6)	64.6 (10.2)	67.4 (12.1)	0.02
**Home dosage of furosemide equivalent, mg/day***	80.0 [40.0, 160.0]	80.0 [40.0, 120.0]	80.0 [40.0, 160.0]	0.64

**Home dosage of loop diuretic**				0.66
Low (0-80 mg furosemide or equivalent	111 (61.3)	55 (65.5)	64 (63.4)	
Middle (81-160 mg furosemide or equivalent	40 (22.1)	21 (25.0)	23 (22.8)	
High, >160 mg furosemide or equivalent	30 (16.6)	8 (9.5)	14 (13.9)	
**Laboratory values at time of first outpatient treatment**				
**Haemoglobin, mmol/L**	7.8 (1.2)	7.5 (1.2)	7.6 (1.1)	0.12
**Sodium, mmol/L**	138.6 (3.7)	138.5 (4.5)	138.7 (3.4)	0.95
**Potassium, mmol/L**	4.3 (0.6)	4.3 (0.6)	4.3 (0.5)	0.72
**Urea, mmol/L**	12.8 [9.3, 17.4]	11.0 [8.5, 15.3]	12.0 [8.4, 17.6]	0.14
**Serum creatinine, umol/L**	140.0 [102.0, 174.0]	117.0 [92.8, 162.8]	125.0 [92.0, 163.0]	0.07
**NT-proBNP, pmol/L**	655.0 [299.0, NA]	355.0 [175.5, 648.8]	215.0 [89.0, 518.0]	<0.001
**eGFR, ml/min/1.73m2**	44.2 (20.1)	45.5 (19.4)	44.1 (20.1)	0.86

Variables are presented as mean (SD), median [IQR] or number (%).
ACE, angiotensin-converting enzyme; ARB, angiotensin-receptor blocker; ARNI, angiotensin receptor-neprilysin inhibitor; BMI, Body Mass Index; COPD, chronic obstructive pulmonary disease; CRT, cardiac resynchronization therapy; DBP, diastolic blood pressure; eGFR, estimated glomerular filtration rate; HF, heart failure; HFrEF, Heart failure with reduced ejection fraction; HFmrEF, heart failure with mildly reduced ejection fraction; HFpEF, heart failure with preserved ejection fraction; ICD, implantable cardioverter-defibrillator; LVEF, left ventricular ejection fraction; MRA, mineralocorticoid receptor antagonist; NT-proBNP, N-terminal pro-B-type natriuretic peptide; OSAS, obstructive sleep apnoea syndrome; RAAS, renin–angiotensin–aldosterone system; SBP, systolic blood pressure; SGLT2, sodium-glucose co-transporter 2; TIA, transient ischemic attack.
**40mg furosemide is equivalent to 1mg bumetanide.*
